# IL-1β promotes glutamate excitotoxicity: indications for the link between inflammatory and synaptic vesicle cycle in Ménière’s disease

**DOI:** 10.1038/s41420-024-02246-2

**Published:** 2024-11-20

**Authors:** Na Zhang, Yongdong Song, Hanyue Wang, Xiaofei Li, Yafeng Lyu, Jiahui Liu, Yurong Mu, Yan Wang, Yao Lu, Guorong Li, Zhaomin Fan, Haibo Wang, Daogong Zhang, Na Li

**Affiliations:** 1 Department of Otolaryngology-Head and Neck Surgery, Shandong Provincial ENT Hospital, Shandong University, Jinan, Shandong China; 2 Shandong Provincial Vertigo Dizziness Medical Center, Jinan, Shandong China; 3 Shandong Medical Health Key Laboratory of Vertigo & Vestibular Medicine, Jinan, Shandong China; 4 Center of Clinical Laboratory, Shandong Second Provincial General Hospital, Jinan, Shandong China

**Keywords:** Cytokines, Immunopathogenesis

## Abstract

Ménière’s disease (MD) is a complex inner ear disorder characterized by a range of symptoms, with its pathogenesis linked to immune-related mechanisms. Our previous research demonstrated that IL-1β maturation and release can trigger cell pyroptosis, exacerbating the severity of the endolymphatic hydrops in a mouse model; however, the specific mechanism through which IL-1β influences MD symptoms remains unclear. This study conducted on patients with MD examined changes in protein signatures in the vestibular end organs (VO) and endolymphatic sac (ES) using mass spectrometry. Gene ontology and protein pathway analyses showed that differentially expressed proteins in the ES are closely related to adhesion, whereas those in the VO are related to synapse processes. Additionally, the study found elevated expression of Glutaminase (GLS) in the VO of MD patients compared to controls. Further investigations revealed that IL-1β increased glutamate levels by upregulating GLS expression in HEI-OC1 cells. Treatment with a GLS inhibitor or an IL-1β receptor antagonist alleviated auditory-vestibular dysfunction and reduced glutamate levels in mice with endolymphatic hydrops. These findings collectively suggest that imbalanced neurotransmitter release and immune responses contribute to the pathology of MD, potentially explaining the hearing loss and vertigo associated with the disease and offering new avenues for therapeutic interventions.

## Introduction

Ménière’s disease (MD) is a complex and highly heterogeneous disorder of the inner ear that affects 3.5–513 per 100,000 every year worldwide [1]. MD is characterized by episodes of spontaneous vertigo, fluctuating sensorineural hearing loss, tinnitus, and/or aural fullness [2]. Patients with MD show the presence of endolymphatic hydrops (EH) in the cochlear duct and vestibular end organs (VO), which may be caused by deficient absorption in the endolymphatic sac (ES) [3]. Several factors have been postulated to be involved in the MD, including viral infections, allergies, genetic factors, and immunity etc. [4–7]. Our previous studies have demonstrated that the maturation and release of IL-1β, triggered by pyroptosis, can exacerbate the severity of the EH mouse model [8, 9]. Nevertheless, the specific mechanism by which IL-1β impacts the symptoms of MD remains to be elucidated.

Previous studies on MD have utilized various approaches to understand its pathogenesis and molecular mechanisms. RNA-sequencing studies of peripheral blood mononuclear cells (PBMCs) support a pro-inflammatory subgroup of MD patients [10]. Cruz-Granados et al. found that there are two clusters of MD patients, one inactive and one Monocyte-driven using single-cell RNA sequencing and single-cell ATAC sequencing analysis of PBMCs [11]. Transcriptome analysis of vestibular organs has shown a close association between MD and neuropathy as well as autoimmune diseases [12]. Proteomic studies on MD patients have revealed overexpression of complement factor H in plasma [13] and disease-specific protein distribution in the perilymph [14], highlighting involvement in inflammatory diseases, energy metabolism, developmental disorders, and cell-to-cell signaling compared to normal perilymph [15]. However, it is noted that using plasma and perilymph in proteomic studies may not fully capture the changes in inner ear tissue. Knowledge of disease-specific molecular changes in the human inner ear is limited due to the challenges posed by the small size and location of inner ear structures within the bony labyrinth. Patients with MD exhibit varying degrees of vestibular dysfunction, yet the exact lesion site and molecular mechanisms underlying the dizziness associated with the condition remain unclear [16].

Excitotoxicity of glutamate is a pathological process that induces neuronal alterations and damage through the excessive release of glutamate and its analogs [17], which occurs in neurological diseases and neurodegenerative diseases such as ischemia, traumatic Alzheimer’s and Multiple Sclerosis [18]. Studies suggest that glutamate plays a crucial role in the occurrence and progression of cochlear synaptic injury [19]. Additionally, inflammation can impact glutamatergic neurotransmission and synaptic integrity [20], with the inflammatory cytokine IL-17 enhancing glutamate excitotoxicity by affecting astrocytic glutamate uptake [21]. Moreover, glutamate can induce the upregulation of pro-inflammatory cytokines, further exacerbating excitotoxicity [22]. While synaptic damage may be linked to MD [23], the direct relationship between primary synaptic damage from glutamate overactivation in vestibular tissue and MD remains unclear.

In this study, we conducted a quantitative proteomic analysis of the VO and ES from patients with MD and vestibular schwannoma (VS), and explored how IL-1β influences glutamate excitotoxicity by increasing the expression of glutaminase (GLS), potentially contributing to the pathogenesis of MD.

## Results

### Protein identification and differential abundant protein (DAP) screening of the human ES and VO

**Fig. 1:**
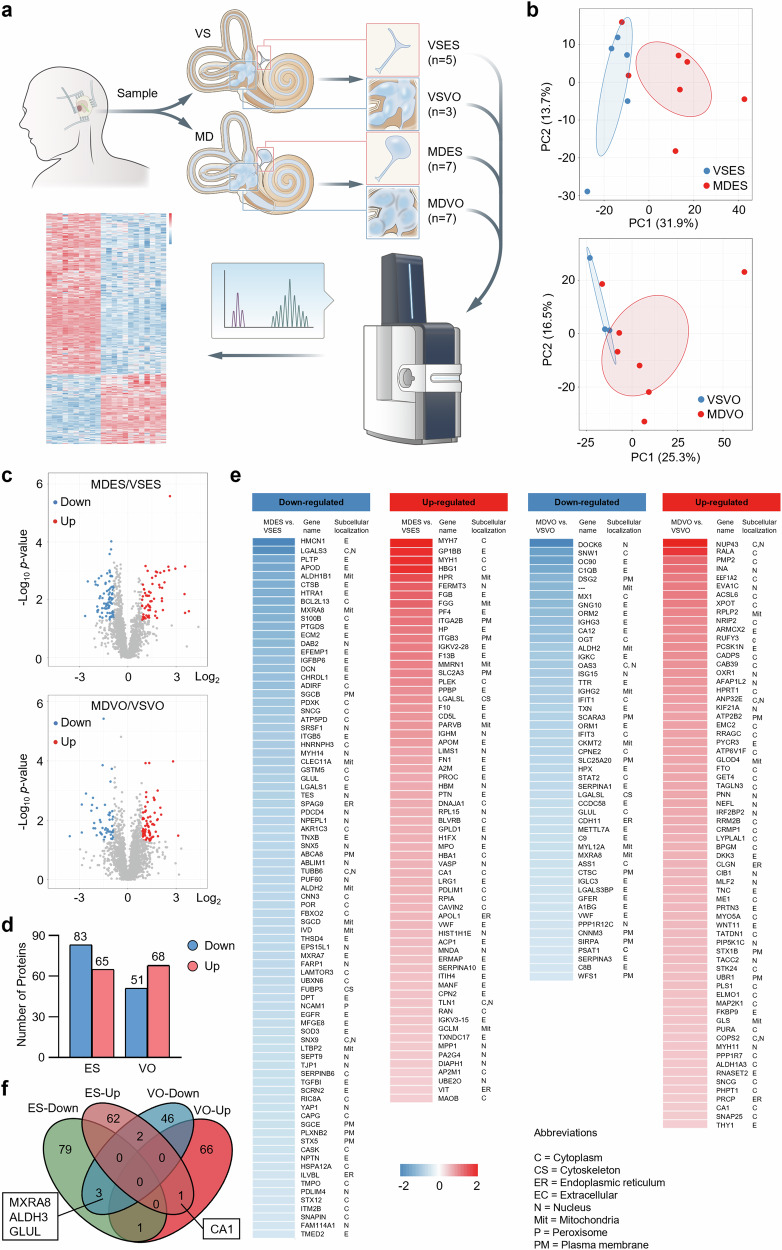
Quantitative proteomics analysis identified different proteins in the ES and VO. **a** A flowchart of the experimental scheme. **b** Protein quantitative principal component analysis results for ES and VO in patients with VS and MD. **c** Volcano plot showing protein expression differences among all proteins identified in the ES and VO samples (red, upregulated proteins; blue, downregulated proteins; gray, unchanged proteins). **d** Histogram representing the distribution of DAPs in the ES and VO. **e** Heat map showing the DAPs and their subcellular localizations. **f** Venn diagrams showing the overlap of DAPs in the ES and VO specimens.

To determine the differences in ES and VO between VS and MD samples, we conducted a quantitative proteomic analysis. Seven VO and ES samples each from 12 patients with MD (MDVO and MDES, respectively) were sampled (Fig. 1a). Three VO and five ES samples from eight patients with VS (VSVO and VSES, respectively) served as the control groups. First, unsupervised principal component analysis (PCA) was performed and the score plot showed that the MD group could be distinguished from the VS group (Fig. 1b). Quantification of individual proteins revealed 148 DAPs in ES (83 upregulated and 65 downregulated) and 119 DAPs in VO (68 upregulated and 51 downregulated) in the MD group that were 1.5-fold different than those in the VS group (*p* < 0.05; Fig. 1c, d; Supplementary Table 3,4]. The upregulated and downregulated proteins as well as their subcellular localization are presented as a heatmap in Fig. 1e. Venn diagrams showed that three DAPs (Matrix remodeling-associated 8 (MXRA8), Aldehyde dehydrogenase 3 (ALDH3), and Glutamine synthetase (GLUL)) were downregulated, whereas Carbonic anhydrase 1 (CA1) was upregulated in both VO and ES in the MD group compared with those in the VS group (Fig. 1f). These results provide a proteomic landscape for ES and VO from MD and VS.

### Functional analysis of DAPs in ES and VO

**Fig. 2:**
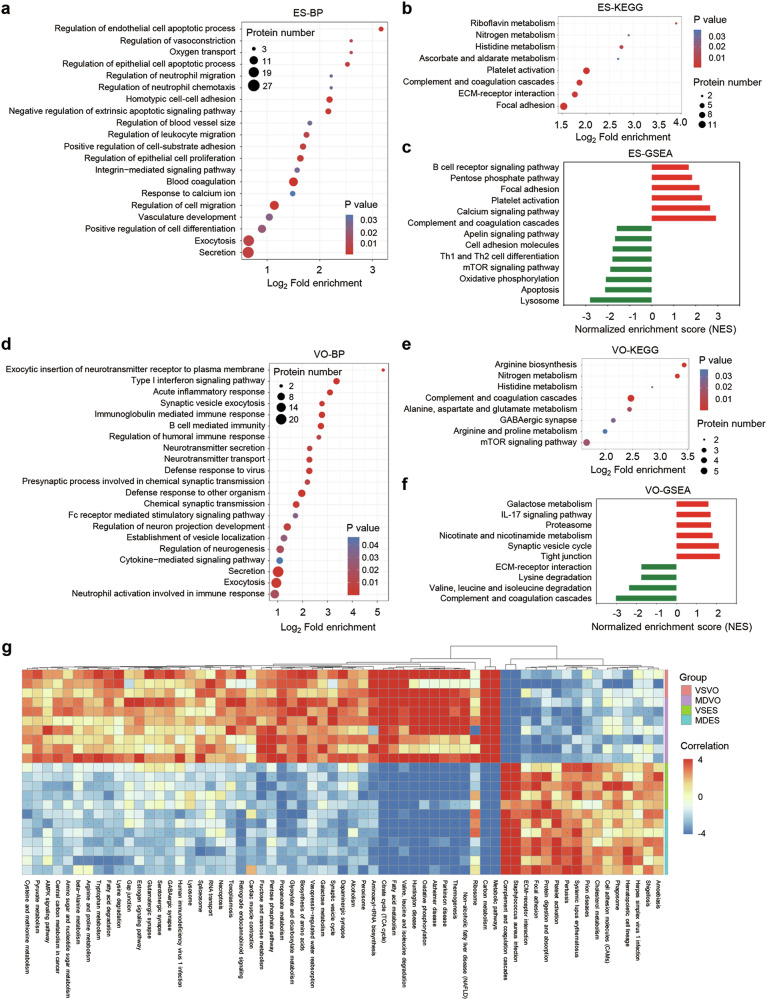
Function enrichment analysis of DAPs in ES and VO. Bubble diagrams representing the enrichment analysis of the DAPs among the VSES and MDES samples enriched in **a** biological process category and **b** KEGG pathway, respectively. **c** Normalized enrichment score for selected representative and significantly enriched gene sets among the VSES and MDES samples. Bubble diagram representing the DAPs enriched among the VSVO and MDVO samples in the **d** biological process and **e** KEGG pathway, respectively. **f** Normalized enrichment score for selected representative and significantly enriched gene sets between the VSVO and MDVO samples. **g** Heatmap of the ssGSEA for ES and VO showing the top 61 most deviated pathways.

To understand the characteristics and functions of the proteins identified in ES and VO, the DAPs between VS and MD samples were annotated by subcellular localization, GO, and KEGG pathways. The subcellular location indicated that the extracellular and cytoplasmic DAPs accounted for 50% of those identified in the ES (Supplementary Fig. 1a). GO terms enrichment of the 148 DAPs revealed the top 20 terms in biological processes, highlighting transport-related processes (e.g., secretion and exocytosis) and adhesion processes (e.g., integrin signaling and cell adhesion) (Fig. 2a). Most DAPs were associated with in the extracellular space (Supplementary Fig. 1b), and were predominantly related to receptor binding and adhesion binding (Supplementary Fig. 1c). KEGG pathway analysis of DAPs revealed significant enrichment in focal adhesion, ECM-receptor interaction, complement and coagulation cascades, and platelet activation (Fig. 2b). PPI network analysis showed the high interactions for pathways related to Rap1 signaling, focal adhesion, endocytosis, and complement and coagulation cascades (Supplementary Fig. 1d), suggesting their potential role in MD pathogenesis. GSEA showed consistent negative or positive enrichment of GO terms associated with cell adhesion, such as focal adhesion and cell adhesion molecules (Fig. 2c).

**Fig. 3:**
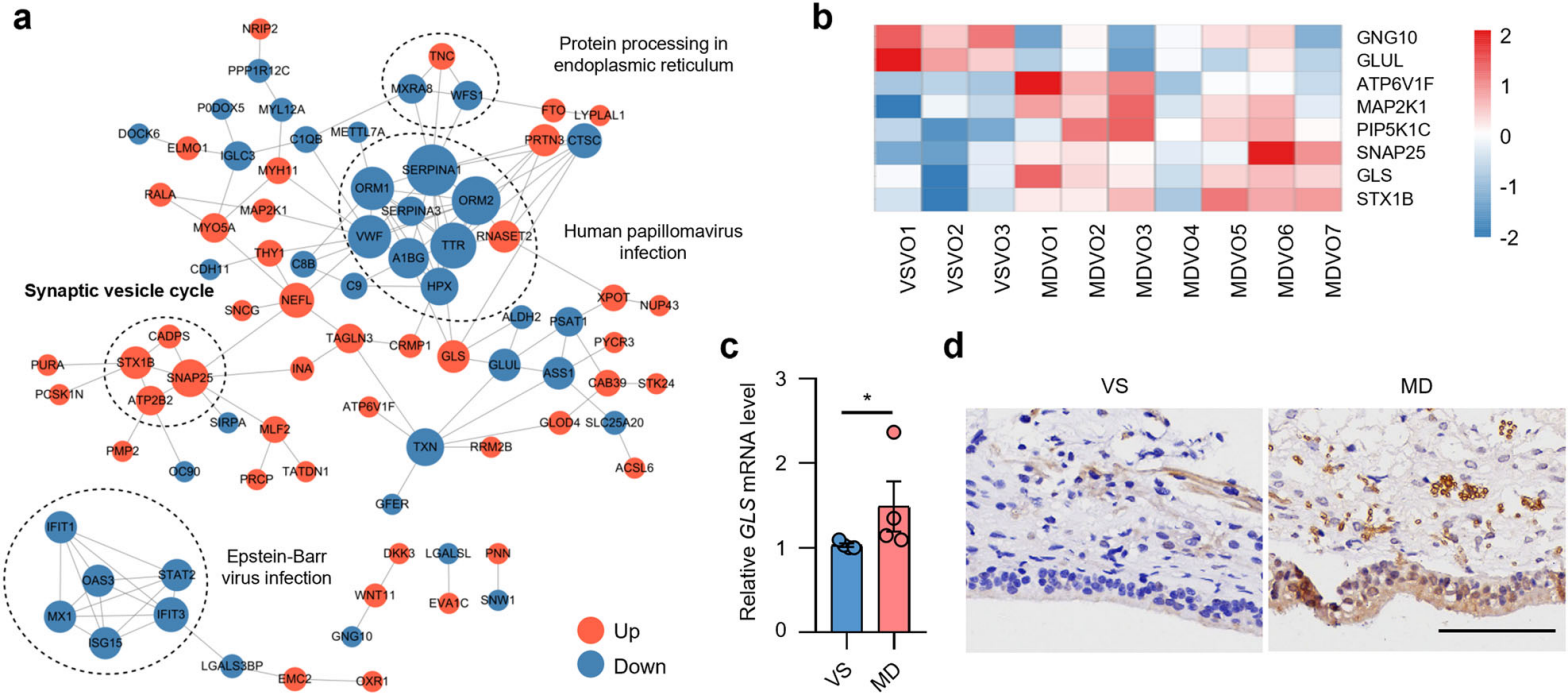
DAPs related to the synaptic vesicle cycle in the VSVO and MDVO. **a** Protein–Protein interaction networks. Different colors represent the differential expression of proteins (blue indicates downregulated proteins and red indicates upregulated proteins). The size of the nodes represents the number of proteins that interact with the DAP. **b** A heatmap showing the DAPs related to the synaptic vesicle cycle. **c** qRT-PCR for *GLS* expression in the VO of VS (n = 4) and MD (n = 4) patients. **d** Representative images of immunohistochemical staining of GLS in the VO of VS (*n* = 3) and MD (*n* = 3) patients. scale bar, 100 μm. *p < 0.05.

For the VO, the cytoplasmic and extracellular proteins accounted for 50% of the DAPs (Supplementary Fig. 2a). The most upregulated proteins were NUP43 (4.244), RALA (3.665), and PMP2 (2.603), while the most downregulated were Dock6 (0.189), SNW1 (0.267) and OC90 (0.267) (Supplementary Table 4). GO enrichment results showed that synaptic processes, including secretion, exocytosis, and neurotransmitter transport, were prominently affected (Fig. 2d). Additionally, biological processes related to immune response, such as neutrophil activation and cytokine signaling, were also significant (Fig. 2d). Most DAPs were associated with in the extracellular space (Supplementary Fig. 2b), and were predominantly related to calcium ion binding and serine-type peptidase activity (Supplementary Fig. 2c). GABAergic synapses were notably enriched in the KEGG database, with most enriched proteins involved in the complement and coagulation cascade pathways (Fig. 2e). Consistent with the GO and KEGG results, the synaptic vesicle cycle was also highlighted in PPI analysis (Fig. 3a), supporting its potential role in the pathogenesis of MD. GSEA showed consistent positive enrichment of GO terms associated with the synaptic vesicle cycle and IL-17 signaling pathway (Fig. 2f). The heatmap further shows that synapse-associated proteins were significantly altered in MD (Fig. 3b). Glutaminase (GLS), a pivotal enzyme in the glutamate synthesis pathway, catalyzes the hydrolysis of glutamine to produce glutamate and involved in the pathogenesis of various neurological disorders [24]. The mRNA and protein levels of GLS in the MDVO was significantly increased compared to the control group (Fig. 3c, d). Collectively, these results indicated that synapse-related transmitter release play an important role in MD pathology.

### The ES and VO have different protein expression patterns and perform different functions

To further reveal the functions of the VO and ES, we compared and analyzed the proteins identified from both tissues. In patients with MD and VS, a total of 19,776 peptides and 2146 proteins in the ES, and 27,650 peptides and 3345 proteins in the VO were identified and quantified (Supplementary Table 5). PCA showed that the protein expression profiles were well separated between the VO and ES, indicating that the two tissues have distinct protein expression patterns (Supplementary Fig. 3a). Quantification of individual proteins showed that 1159 DAPs had significantly different expression (830 upregulated and 329 downregulated) in the VO group based on 1.5-fold changes vs. the ES group (Supplementary Fig. 3b–d). The DAPs were mainly distributed in the cytoplasm, extracellular space, and nucleus (Supplementary Fig. 3e). The ssGSEA showed positive enrichment of metabolic pathways and negative enrichment of complement and coagulation cascades in the VO compared to ES (Fig. 2g). The GO and KEGG enrichment further showed that the highly expressed proteins in the ES were mainly involved in secretion by cells and exocytosis, complement and coagulation cascades and cell adhesion (Supplementary Fig. 3f,h), while those in the VO were mainly related to metabolic processes (Supplementary Fig. 3g, i). These findings highlight distinct protein expression patterns and suggest different roles of ES and VO in MD pathogenesis.

### Anakinra alleviates the phenotype of EH mice and reduces the level of glutamate in the inner ear by downregulating GLS

**Fig. 4:**
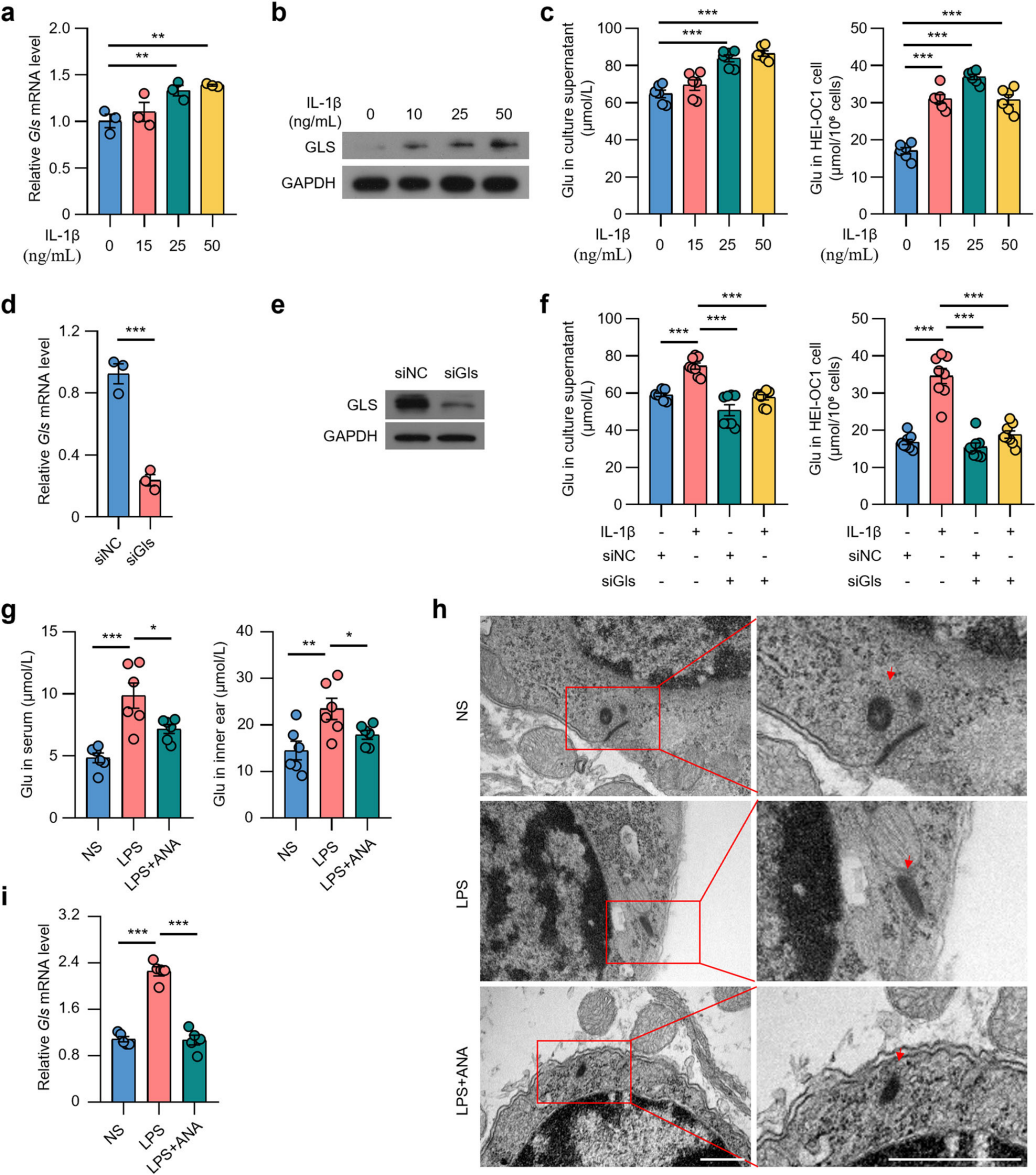
Anakinra alleviates the phenotype of EH mice and reduces the level of glutamate in the inner ear by downregulating GLS. **a** qRT-PCR for *Gls* expression in HEI-OC1 cells (*n* = 3) stimulated with IL-1β (10, 25, 50 ng/mL) for 24 h. **b** Representative images of western blotting detecting GLS in HEI-OC1 cells (*n* = 3) stimulated with IL-1β (10, 25, 50 ng/mL) for 24 h. GAPDH was used as a loading control. **c** Glutamate level in HEI-OC1 cells (*n* = 6) stimulated with IL-1β (10, 25, 50 ng/mL) for 24 hours. **d** qRT-PCR for *Gls* expression in HEI-OC1 cells (*n* = 3) treated with or without siGls. **e** Western blotting to analyze GLS expression in HEI-OC1 cells (*n* = 3) treated with or without siGls. GAPDH was used as a loading control. **f** Glutamate level in HEI-OC1 cells (*n* = 6) stimulated with IL-1β and/or siGls for 24 h. **g** Glutamate level in serum and inner ear of mice (*n* = 6) untreated or pretreated treated with Anakinra (10 mg/kg, i.p.) and challenged with LPS (10 mg/kg, p.a.) or equivalent saline for 3 consecutive days. **h** Transmission electron microscopy showing the ultrastructure of VO in mice (*n* = 3) untreated or pretreated treated with Anakinra and challenged with LPS or equivalent saline. Arrows mark the synapse. Scale bars,500 nm. **i** qRT-PCR for *Gls* expression in mice (*n* = 5) untreated or pretreated treated with Anakinra and challenged with LPS or equivalent saline. *p < 0.05, **p < 0.01, ***p < 0.001.

We then explored the role of IL-1β in regulating glutamate synthesis. As shown in HEI-OC1 cells, IL-1β facilitated the expression of GLS mRNA and protein (Fig. 4a, b), and increased glutamate levels in both the culture supernatant and cells (Fig. 4c). Conversely, the downregulation of GLS using siRNA inhibited the elevated glutamate levels induced by IL-1β (Fig. 4d–f). Anakinra, IL-1β receptor antagonist, was administered to EH model mice, resulting in alleviation of hearing loss and vestibular dysfunction (Supplementary Fig. 4a–d). Furthermore, Anakinra significantly reversed the LPS-induced elevation of glutamate in the mouse inner ear and serum (Fig. 4g), TEM sections revealed that ribbons in vestibular hair cells of LPS-treated mice show abnormal synapses with extremely swollen presynaptic ribbon structure, and ribbons were “free-floating” in the cytoplasm far away from the active site (Fig. 4h). Additionally, Anakinra was found to inhibit synaptic damage (Fig. 4h), and reversed the LPS-induced upregulation of *Gls* in the inner ear (Fig. 4i). In conclusion, these findings collectively suggest that IL-1β upregulates of GLS expression in the inner ear, leads to increased glutamate levels, thereby impairing the function of the auditory-vestibular system.

### Inhibition of GLS alleviates the phenotype and reduces the level of glutamate in the inner ear of EH mice

**Fig. 5:**
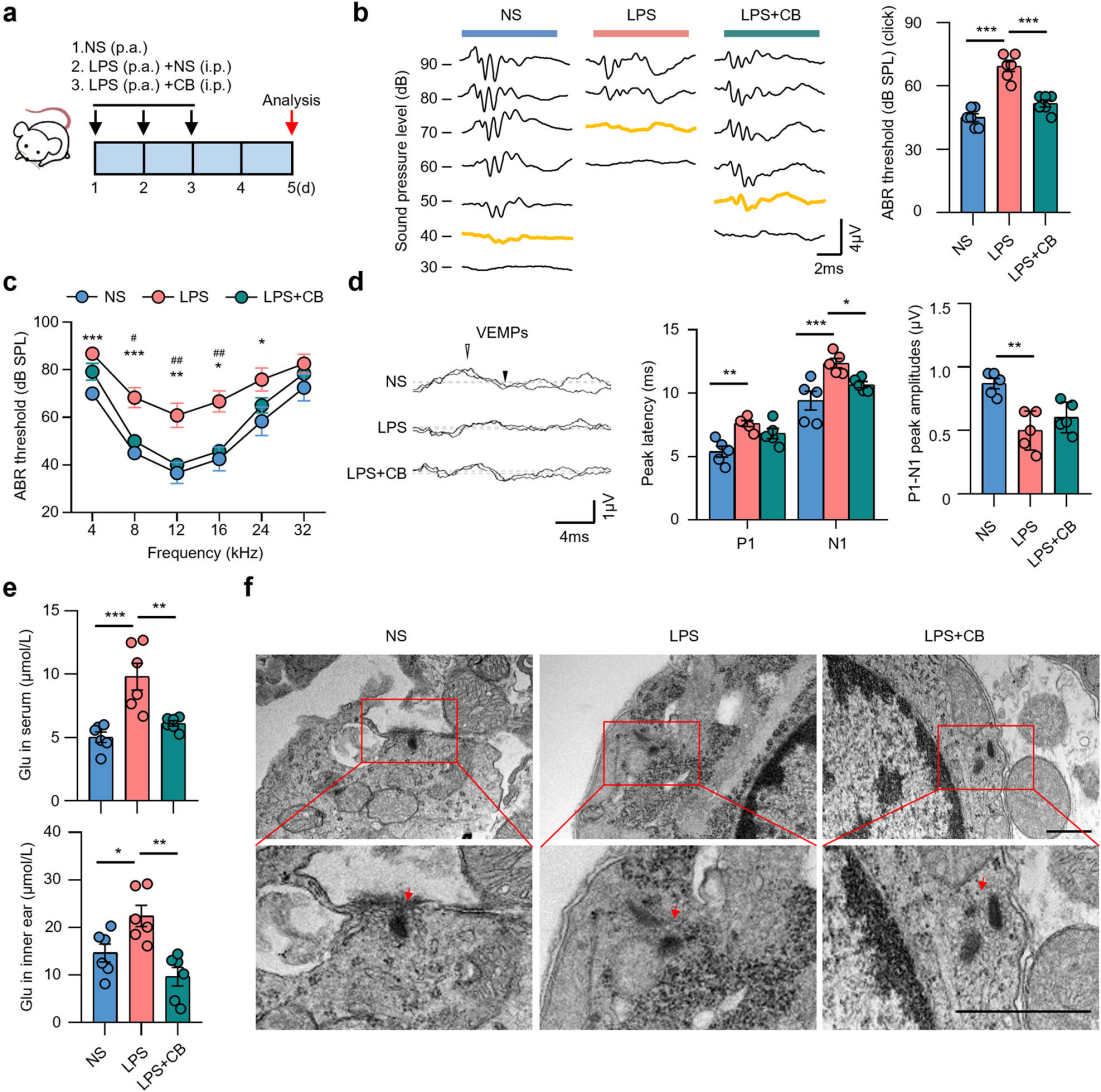
Inhibition of GLS alleviates the phenotype of EH mice and reduces the level of glutamate in the inner ear. **a** Mice were subjected to LPS (10 mg/kg, p.a.) and concurrently treated with the GLS inhibitor CB-839 (28 mg/kg, i.p.) in the meantime for 3 consecutive days, then analyzed at 5 days. **b** Representative serial ABR wave recordings and thresholds in response to click sounds (*n* = 6). **c** ABR thresholds in response to tone pip across all frequencies (*n* = 6). * significant difference compared with NS and LPS; # significant difference compared with LPS and LPS + CB. **d** Representative click-evoked VEMP waves, P1-N1 peak amplitudes, and the P1 (white triangle) and N1 (black triangle) peak latencies of VEMPs at 100 dB nHL (*n* = 5). **e** Glutamate level in serum and inner ear of mice (*n* = 6). **f** The ultrastructure of VO in mice (*n* = 3) was observed by transmission electron microscopy. Arrows mark the synapse. Scale bars, 500 nm. * or # *p* < 0.05, ** or ^##^*p* < 0.01, *** or ^###^*p* < 0.001.

Subsequently, mice were treated with the GLS inhibitor CB-839 to elucidate the impact of GLS on EH mice (Fig. 5a). The results showed that click-induced ABR threshold were considerably higher in LPS mice compared to saline group, these effects were significantly reversed by CB-839 therapy (Fig. 5b). For tone burst-induced ABR, LPS induced the threshold increases in the frequency range from 8 to 24 kHz, and mice treated with CB-839 reversed threshold increases in the frequency range from 8 to 16 kHz caused by LPS (Fig. 5c). For vestibular function, the LPS mice showed increased latency of P1 and N1, and decreased P1-N1 amplitudes compared to those control mice (Fig. 5d). Indeed, CB-839 treatment decreased the latency of N1 (Fig. 5d). Furthermore, CB-839 significantly reversed the LPS-induced elevation of glutamate in the mouse inner ear and serum (Fig. 5e), and inhibited synaptic damage as shown in TEM sections (Fig. 5f). Together, these data showed that CB-839 alleviated damage to auditory-vestibular function and reduces the level of glutamate in LPS-induced EH mouse model.

## Discussion

MD is one of the most common disorders in the field of otolaryngology. Previous proteomic studies have utilized plasma [13, 25] and perilymph [15] samples from MD patients to investigate molecular changes associated with the disease. However, these studies have been limited in revealing alterations in inner ear tissues. In this study, we analyzed the proteomic patterns of VO and ES of patients with MD and VS, which revealed the following: (1) Compared with the VS group, there were 148 DAPs in the MDES, and the DAPs were closely related to adhesion; (2) here were 119 DAPs in the MDVO, and the DAPs were closely related to synapse-related processes; (3) IL-1β increased glutamate levels by promoting GLS expression in HEI-OC1 cells, (4) GLS inhibitor CB-839 or the IL-1β receptor antagonist Anakinra alleviated the phenotype and reduced the glutamate levels in the EH mice. Therefore, our data provide comprehensive insights into the global molecular landscape of MD and suggest that the synaptic vesicle cycle is and dysregulated immune responses involved in MD and partially accounts for hearing loss and vertigo.

The ES, a crucial non-sensory inner ear organ, is responsible for electrolyte homeostasis, endolymph resorption, and immune defense [26, 27]. Deficient absorption in the ES is a pathological cause of EH [3], and the damage of the ES epithelium is linked to MD [28], making the ES a primary affected site in MD [29]. Animal models that alteration in endolymph production and absorption have been used to explain the histopathological and clinical features of MD [30–32]. The vestibular system, essential for spatial orientation, head movement and balance, comprises semicircular canals that sense angular acceleration and otolith organs (saccule and utricle) that detect linear acceleration [33]. Impaired otolith organ function [34] and neuroepithelial degeneration in the VO were noted in MD [35–37]. Our study identified and quantified proteins in MD and VS, revealing different expression patterns and functions: the highly expressed proteins in the ES were involved in complement and coagulation cascades and cell adhesion, whereas those in the VO were metabolism-related proteins. The DAPs in the ES were mainly related to adhesion and transport, while those in the VO were related to neurotransmitter release. Pathogenic sites in MD may involve multiple organs, with ES and VO potentially playing a role in the disease’s pathophysiology. ES and VO functions may interact, impacting the development of MD. However, the causal relationship between ES and VO lesions requires further investigation.

The bioinformatics analysis conducted in the study identified key factors for MD in the VO, highlighting neurotransmitter pathways and the synaptic vesicle cycle as crucial elements. In a healthy inner ear, synaptic transmission such as neurotransmitter pool size, vesicular release probability, and postsynaptic channel conductance are optimized for proper neural network function [38]. Genetic mutations and environmental factors like acoustic overstimulation, can cause hearing loss by damaging hair cells or degrading synapses [39]. However, the precise mechanisms underlying hearing loss are not fully understood. Hair cells in the VO are also mechanosensitive and release graded amounts of glutamate into the vestibular ganglion cells depending on the deflection of stereocilia [33]. Our study observed elevated levels in neurotransmitter release-related proteins (SNAP25 and STX1B [40, 41]) and glutamate synthesis-related protein (GLS [24]), and decreased in glutamate clearance-related protein (GLUL [42]) in MDVO. RNA-seq studies have reported the molecular mechanisms underlying MD and neuropathy [12]; therefore, we suggest that increased release and decreased breakdown of glutamate neurotransmitters in MD leads to vertigo episodes, and excitotoxicity further leads to the swelling and degeneration of nerve endings, which result in hearing loss.

Previous studies have proposed that immune responses and immune-related processes are critical in MD [4, 43]. Functional enrichment analysis of DAPs in the VO indicated that numerous dysregulated proteins were linked to various immune response, such as B cell-mediated immunity, immunoglobulin-mediated immune response, response to type I interferon, IL-17 signaling pathway, and regulation of humoral immune response. additionally, the complement and coagulation cascades were enriched in KEGG in the ES. Notably, these pathways could potentially serve as novel targets for the treatment of MD.

Our previous research demonstrated that IL-1β plays a pivotal role in the pathogenesis of MD [8, 9]. Several studies have indicated that MD patients exhibit high basal levels of IL-1β in serum [11, 44]. IL-1β has been shown to influence glutamate release, uptake, and receptor expression, hereby affecting synaptic transmission and neuronal excitability. In autoimmune encephalomyelitis, IL-1β participates in the abnormal discharge of central nervous system neurons in a dose-dependent manner [45]. In macular degenerative diseases, IL-1β inhibits glutamate transport, leading to excessive accumulation of glutamate in the retina and inducing degeneration of rod cells [46]. Furthermore, IL-1β also increase glutamate synthesis by promoting the expression of GLS in neurons [47]. Here, we found that GLS expression was increased in VO of MD patients, and IL-1β facilitated the increase of glutamate levels by promoting the expression of GLS in HEI-OC1 cells. Furthermore, proteomic data showed that MXRA8, ALDH3 and GLUL were downregulated and CA1 upregulated. As a key enzyme in the glutamate-glutamine cycle, the decreased expression of GLUL leads to the accumulation of glutamate [48], which may indirectly affect the expression of GLS. Given the pH sensitivity of GLS, the pH alterations caused by the upregulation of carbonic anhydrase 1 (CA1) may modulate the activity of GLS [49]. MXRA8 [50] and ALDH3 [51] may affect GLS expression by regulating cell-matrix interactions and cell redox, respectively. Additionally, GLS inhibitor CB-839 and inhibitor of the IL-1β receptor Anakinra alleviated the hearing loss and vestibular dysfunction, and reduced the level of glutamate in the endolymphatic hydrop mice. Therefore, we propose local IL-1β in the inner ear may promote glutamate synthesis through upregulation of GLS, induce neurodegeneration, and thus participate in the development of MD. Our previous studies demonstrated that anakinra contributes to a promising therapeutic approach in a murine model of EH, by restricting EH, alleviating audio-vestibular function, inhibiting inflammation of the inner ear and protecting the cochlear nerve [9]. The present study used new animals and found that anakinra significantly reversed the LPS-induced elevation of glutamate in the mouse inner ear and serum, inhibited synaptic damage, and reversed the LPS-induced upregulation of GLS in the inner ear. Based on our previous studies, we further verified the crucial role of IL-1β and glutamate excitotoxicity in the pathogenesis of MD, which may serve as potential therapeutic targets.

There are limitations to our study. MD is a complex disorder involving multiple mechanisms, including genetic and immune factors. The human vestibular tissue is precious resource, as it is only obtained via surgery [52]; hence, the sample size in our proteomics studies is limited, and the patients were not clustered. The GO and KEGG analysis of DAPs revealed an association between MD and synaptic vesicle cycle, but do not support any specific immune process.

Overall, the data from our study offer a comprehensive understanding of the molecular landscape of MD, highlighting the involvement of dysregulated immune responses and the synaptic vesicle cycle in the pathogenesis of MD. While advancements have been made in comprehending the molecular aspects of MD, substantial gaps persist in understanding the precise mechanisms leading to vertigo attacks and hearing fluctuations in MD. Further research, particularly focusing on neurotransmitter release, the cause of endolymphatic hydrops, and pathology of inner ear, is imperative to advance our understanding of MD pathogenesis and develop more effective treatment strategies.

## Materials and methods

### Patients and tissue samples

Patients with MD were recruited based on the 2015 diagnostic criteria for MD [53]. A total of 16 patients with MD aged 46–74 years (average age = 58.8 ± 6. 2 years) and 14 patients with VS aged 45–74 years (average age = 54.9 ± 8.2 years) at Shandong Provincial ENT Hospital from December 2018 to November 2023 was enrolled. The demographic and clinical features of the patients with MD are described in Supplementary Table 1. Written informed consent was obtained from all the participants. The study protocol was conducted according to the principles of the Declaration of Helsinki, revised in 2013 for investigation with humans, and approved by the Ethics Committee of Shandong Provincial ENT Hospital (IRB ID: XYK-20180603).

Ampullae, maculae, and ES were sampled during labyrinthectomy. For Liquid chromatography-mass spectrometry analysis (LC–MS/MS) analysis, the samples were homogenized in lysis buffer (8 M urea, 1% protease inhibitor cocktail, Calbiochem), followed by sonication on ice. After centrifugation at 12,000 × *g* at 4 °C for 10 min, the supernatant was collected and the protein concentration was measured using BCA kit (Beyotime Biotechnology, Shanghai, China) according to the manufacturer’s instructions. For frozen sections, the collected vestibular tissue samples were fixed with 4% paraformaldehyde.

### LC–MS/MS

For the LC–MS/MS analysis, the protein solution was digested by trypsin and dissolved in 0.1% formic acid, after which they were loaded onto a reversed-phase analytical column. They were subsequently eluted on an EASY-nLC 1000 UHPLC system with a gradient mobile phase B (0.1% formic acid in acetonitrile) from 6% to 23% over 26 min, 23% to 35% over 8 min, to 80% over 3 min, and then held at 80% for the last 3 min, at 400 nl/min. The resulting peptides were subjected to Capillary source, followed by LC–MS/MS in tims-TOF Pro (Bruker, Karlsruhe, Germany). The electrospray voltage was 1.4 kV. Precursors and fragments were analyzed using the Orbitrap with MS/MS scanning range from 100 to 1700 *m*/*z*. Ten fragment spectra were obtained per cycle and dynamic exclusion was set to 24 s. The automatic gain control (AGC) was 5E4. The fixed first mass was set at 100 *m*/*z*.

### Protein identification and quantification

Proteins were identified in the Uniport database using the MaxQuant search engine (version 1.6.6.0), and the database search was Homo_sapiens_9606_SP_20191115 (78139 entries) for VO, and Human_SwissPort (20422 entries) for ES. For search parameters, the digestion method was set to Trypsin/P, the number of missing cleavages was four, and the mass tolerance for the first search and main search were 70 ppm. The fragment mass tolerance was 0.04 Da. For fixed modifications, carbamidomethyl on Cys was selected; for variable modifications, acetylation modification and oxidation of Met were selected. Other conditions were as follows: FDR < 1%, PSM < 1%, and minimum score for modified peptides >40. Protein quantification was based on the median of the unique peptides of the protein. By calculating the relative expression ratio of the target protein to the reference protein for the sample, the differences in protein abundance between the two groups were compared. Finally, the median protein ratio was used to normalize the peptide ratios.

### Bioinformatics analysis

To analyze the identified differentially expressed proteins, Gene Ontology (GO) (InterProScan, version 5.14–53.0) terms including cellular components, molecular functions, and biological processes were used. The identified protein domain functional descriptions were annotated using InterproScan (version 5.14–53.0). The KEGG database (KAAS, version 2.0; KEGG Mapper, version 2.5) was used to annotate the protein pathways. Wolfpsort (version 0.2) was used to predict protein subcellular localization. For the functional enrichment of GO, pathway, and protein domain analyses, a two-tailed Fisher’s exact test was used to test the enrichment of target proteins, and a corrected *p* < 0.05 was considered significant. Protein cluster analysis was performed using the ComplexHeatmap R package (R, version 3.4). The filtered P matrix was transformed using −log10 (*P*-value). Enrichment-based clustering was visualized using the R package heatmap (version 2.03). The protein–protein interaction (PPI) based on the STRING database was visualized using R package networkD3 (version 0.4). Cytoscape (version 3.10.0) was used to rank the genes within this network based on their degree centrality values. Gene set enrichment analysis (GSEA) was based on the algorithm developed by the Broad Institute (https://www.gsea-msigdb.org/gsea/index.jsp). For single sample GSEA (ssGSEA), the relative quantification values of VO and ES combined proteins and the KEGG annotation data of the project were used as inputs and analyzed using ssGSEA2.0 (https://github.com/broadinstitute/ssGSEA2.0).

### Establishment of animal models and intervention

Wild-type male C57BL/6 mice (6–8 weeks old, weighing 17–25 g) were purchased from the Animal Center of Shandong University, housed in a temperature-controlled (20–22 °C) room with a 12/12 h light/dark cycle and had free access to food and drinking water. All study protocols were approved by the Animal Care Committee of Shandong University and conformed to the Guidelines for the Care and Use of Laboratory Animals of the National Institutes of Health.

Age and sex-matched mice were randomly divided into experimental and control groups. Sample sizes were based on preliminary experiments. C57BL/6 mice were subjected to a model of EH induced by lipopolysaccharide (LPS). Briefly, mice were challenged with LPS (2 mg/kg, L2880, Sigma) in saline by postauricula injection (p.a.) once a day for three days [8], while control groups were p.a. injected with equivalent 0.9% NS. To assess the role of IL-1β in LPS-induced EH, mice were i.p. injected with the IL1-receptor antagonist anakinra (10 mg/kg, HY-108841, MCE) or an equivalent NS 30 min prior to LPS challenge [9]. To assess the impact of GLS inhibition, C57BL/6 mice were subjected to LPS induction and concurrently treated with the GLS inhibitor CB-839 (GLS1 inhibitor, 28 mg/kg, HY-12248, MCE) intraperitoneal injection (i.p.). In all cases, hearing and vestibular function were evaluated on day 5 following the initial LPS injection. Pathology and function analysis for animals were performed in single blinded.

### Auditory brainstem response (ABR)

ABR measurements were performed as previously described [54]. ABR responses were measured with a tone pip stimulus at 4, 8, 12, 16, 24, and 32 kHz using a TDT system 3 (Tucker-Davis Technologies, Alachua, FL, USA) with 1024 stimulus repetitions per record in a sound isolation booth. Briefly, mice were anesthetized with a mixture of xylazine (10 mg/kg) and ketamine (100 mg/kg) by i.p. injection. Needle electrodes were inserted into the subcutaneous tissue at the vertex (recording electrode), infra-auricular mastoid region of the ipsilateral ear (reference electrode), and back (ground electrode). The sound level started at a 90 dB sound pressure level (SPL) and then decreased by 5 dB to the acoustic threshold. The ABR threshold was determined at each frequency, which refers to the minimal SPL that resulted in a reliable ABR recording with one or more distinguishable waves clearly identified by visual inspection. This process was repeated for low SPLs near the threshold to ensure waveform consistency.

### Vestibular-evoked myogenic potential (VEMP)

Click-evoked VEMP recordings were initiated with simultaneous recording of electromyography potentials following anesthesia. A custom-made holder previously reported [55] was used for these recordings. The necks of the mice were hyperextended and stabilized with a suspension wire fixed behind the front teeth. Mice were kept in a prone position with an elevated head and free legs. During recording, a platinum needle electrode was inserted into the cervical extensor muscles and a reference electrode was placed on the cervico-occipital region at the midline. The ground electrode was then repositioned. The VEMP test was performed on each animal at a stimulus intensity of 100 dB nHL. The response threshold was the lowest threshold for the appearance of a waveform, with repeatability verified by consecutive runs (>3 times). Finally, the latencies and amplitudes of the negative and positive peaks were measured.

### Rotarod test

The mice were placed on an electric rotating rod (ZH–600 B, Huaibei Zhenghua Biological Instruments Co., Ltd.) at a maximum speed of 30 rpm. The speed was then gradually increased to 30 rpm for 2 min. Each mouse was trained 2 times per day for 3 days and tested twice. Thereafter, the average time taken to fall off the rotarod in the two trials was recorded for analysis.

### Vestibular ocular reflexes (VOR)

VOR tests were performed as previously described [56]. Briefly, mice were implanted with a noninvasive animal immobility setup. Horizontal eye position signals were recorded using a binocular VOG-based VFT system provided by Prof. Fangyi Chen’s team at the Southern University of Science and Technology. An infrared camera equipped with a zoom lens (MI, China) was mounted on the translation stages at a 45° angle to the anteroposterior axis of the mouse. Illumination for video recording was achieved using two near-infrared light-emitting diode lamps (940 nm, Chundaxin®, China) attached to the camera. The eye tracker tracked the region of interest (ROI) at a speed of 60 frames per second. The ROI, which contained the pupil, in each frame was automatically selected using a template-matching method. Subsequently, an ellipse fit was applied to define the center of the pupil, and the horizontal component was extracted from the eye movement recordings. Calibration was performed to convert the acquired translational distance to the eye rotation angle. Following calibration, a series of linear accelerations were recorded. To measure the VOR responses, horizontal rotations were recorded at 20°/s (0.2, 0.5, 0.8, 1.0, 1.6, 3.2 and 5.0 Hz). The exported eye-location data underwent Fourier transformation using MATLAB 2016b software to obtain the amplitude data for eye movement. The VOR gain was calculated as the amplitude ratio between the response and the stimulus. VOR measurements were performed by investigators blinded to the mouse study.

### Cell lines, cell culture, and treatments

HEI-OC1 cells were cultured in high‐glucose Dulbecco’s modified Eagle medium (DMEM; Gibco, Grand Island, NE, USA) with 10% fetal bovine serum (Gibco) at 33 °C in a humidified incubator containing 10% CO2. In the experiments, HEI-OC1 cells were stimulated with IL-1β (10, 25, 50 ng/mL, HY-P7073A, MCE) for 24 h.

One pair of siRNAs targeting the mouse *Gls* gene were used to silence GLS expression in HEI-OC1 cells. The sequences of the *Gls* siRNAs used were as follows: sense, 5′- GAGGGAAGGUUGCUGAUUATT-3′, antisense, 5′- UAAUCAGCAACCUUCCCUCTT-3′. A nontargeting scramble siRNA was used as a negative control treatment (GenePharma, Shanghai, China). In brief, siRNA and Lipofectamine RNAiMAX (Invitrogen, Carlsbad, CA, USA) were added to Opti-MEM (Invitrogen). Solutions of siRNA and Lipofectamine RNAiMAX were then mixed and incubated at room temperature. Equal volumes of the mixture were added to the culture plates and cultured for 48 hours.

### Immunohistochemistry analysis

The inner ear specimens were obtained from patients and were fixed with 4% paraformaldehyde overnight at 4°C. The samples were dehydrated by successive treatments with 15%, 20%, and 30% sucrose in phosphate-buffered saline, and embedded in OCT compound (Tissue‐Tek, Sakura Finetek, Torrance, USA). The 5-μm specimens were sectioned using a cryostat (Leica CM 1850, Nussloch, Germany) and stored at -80°C. A DAB Detection Kit (Streptavidin-Biotin) kit (SP-9000-D; ZSGB-BIO, Beijing, China) was utilized for assessing the expression of the VO. Briefly, the endogenous peroxidase activity was inhibited by treating the section with 0.3% hydrogen peroxide for 30 minutes. To prevent nonspecific binding, the sections were incubated with 10% normal goat serum for 20 min. The sections were then stained with anti-GLS (1:200, sc-74430; Santa Cruz, CA, USA) overnight at 4 °C. Following a rinse with PBS and incubation with biotinylated anti-rabbit or anti-mouse IgG for 30 minutes, the sections were incubated with DAB complex for 1 min. The sections were counterstained with hematoxylin, dehydrated, and omitted with Neutral balsam (G8590; Solarbio, Beijing, China). Images were performed using a Leica microscope.

### Enzyme-linked immunosorbent assay (ELISA)

The inner ear of mice were harvested, immediately snap-frozen in liquid nitrogen, and stored at −80 °C. Subsequently, inner ear tissues were placed in lysing buffer and homogenized. The homogenate was then centrifuged, and the supernatant was collected for ELISA analysis. Enzymes and standard glutamate (ab83389; Abcam, Cambridge, MA, USA) were used to determine glutamate concentrations of serum and inner ear according to manufacturer’s instructions. Microplate reader (Bio-RAD) was used to detect the signals at 450 nm.

### Transmission electron microscopy (TEM)

The maculae of mice were harvested, immediately fixed using a 3% glutaraldehyde fixative solution and 1% osmic acid, dehydrated, infiltrated, and embedded in Epon 812. Subsequently, ultrathin radial sections were stained with lead citrate and uranyl acetate before being examined under a transmission electron microscope (JEOL-1200EX, Japan).

### Quantitative real-time PCR (qRT-PCR)

The total RNA was extracted using an RNA extraction kit (RNeasy Mini QIAcube Kit, QIAGEN, Hilden, Germany). The relative expression level of RNA was measured by qRT-PCR using an Eppendorf AG 22331 PCR machine (Eppendorf, Hamburg, Germany). The qRT-PCR reaction mixture included 2 × SYBR Green Premix EX Taq (RR42LR; Takara Biotechnology, Shiga, Japan), cDNA template, forward and reverse primers, and deionized water. The qRT-PCR parameters included an initial denaturation step at 95 °C, followed by 40 cycles of denaturation at 95°C, annealing at 60°C, and finally elongation at 72°C. The specificity of each PCR was confirmed using melting curve analysis. Primers used in this study are listed in Supplementary Table 2. *GAPDH* was used to normalize target mRNA expression. The fold change for each gene was determined using the comparative Ct method [54].

### Western blot

Total protein extraction of HEI-OC1 cells was conducted using cold RIPA lysis buffer supplemented with a protease inhibitor cocktail. Equal amounts of protein samples were heat-denatured at 99°C for 10 min and subsequently separated via 10% SDS-PAGE gel electrophoresis. The proteins were then transferred onto polyvinylidene difluoride membranes (Millipore). These membranes were blocked in 5% skim milk for 1 hour before being incubating with anti-GLS (1:100, sc-74430; Santa Cruz) and anti-GAPDH (1:8,000, ab8245; Abcam). The following day, the membranes were exposed to HRP-conjugated secondary antibodies (1:10,000, 115-035-003, Jackson) for 1 h. Finally, protein signals were visualized using an ECL kit (Millipore). All blocking, incubation, and washing steps were carried out using Tris-buffered saline with 0.05% Tween 20.

### Statistical analysis

All data analyses were single blinded. The data were statistically analyzed using SPSS 21.0 statistics software (SPSS Inc., Chicago, IL, USA), except for bioinformatics analysis. Bioinformatics-related statistical software and methods are described in the bioinformatics analysis section. Quantitative data are presented as mean ± standard error of the mean (SEM) and analyzed by Student’s unpaired *t*-test or one-way analysis of variance (ANOVA). A *p* < 0.05 was considered statistically significant.

## Supplementary information

Supplementary Figure

Supplementary Table 1

Supplementary Table 2

Supplementary Table 3

Supplementary Table 4

Supplementary Table 5

original western blots

## Data Availability

The mass spectrometry proteomics data have been deposited to the ProteomeXchange Consortium [57] via the iProX partner repository [58] with the dataset identifier IPX0005380000.
