# Supplementary material for: IL-1β promotes glutamate excitotoxicity: indications for the link between inflammatory and synaptic vesicle cycle in Ménière’s disease
Source: Cell Death Discov. 2024 Nov 20;10:476. doi: 10.1038/s41420-024-02246-2 (PMC11579495; doi:10.1038/s41420-024-02246-2)
Supplement: Supplementary file 1 — Supplementary Figure [file 41420_2024_2246_MOESM1_ESM.docx]

**
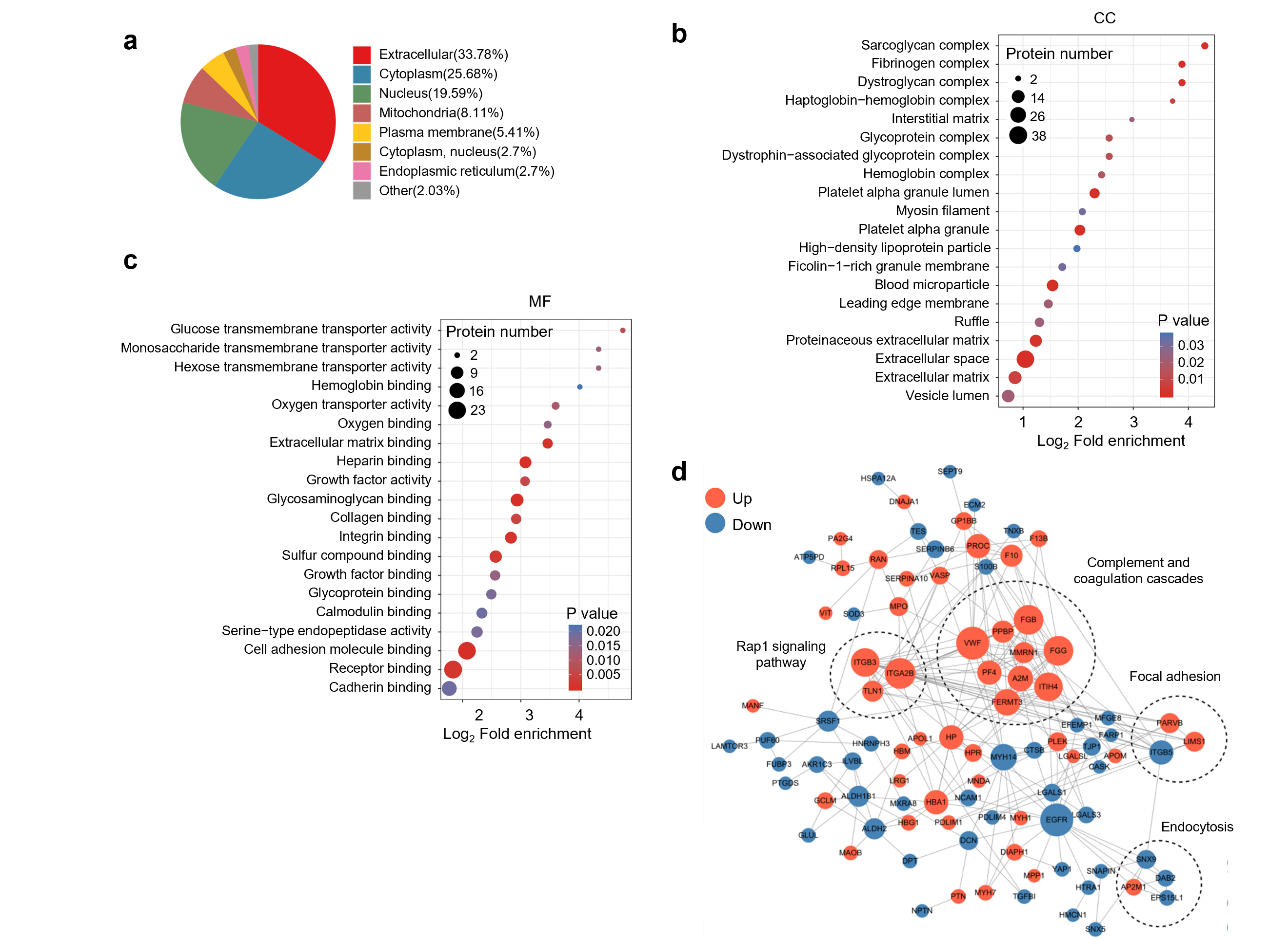
 Supplementary Fig. 1 Function enrichment analysis of DAPs in VSES and MDES**

**a** The subcellular location of DAPs observed in VSES and MDES samples. Bubble diagram representing the DAPs of among VSES and MDES enriched in the (**b**) cellular components and (**c**) molecular functions, respectively. **d** Protein-Protein Interaction Networks. Different colors represent the differential expression of proteins (blue and red indicate downregulated and upregulated proteins, respectively). The size of the nodes represents the number of proteins interacting with the DAPs.


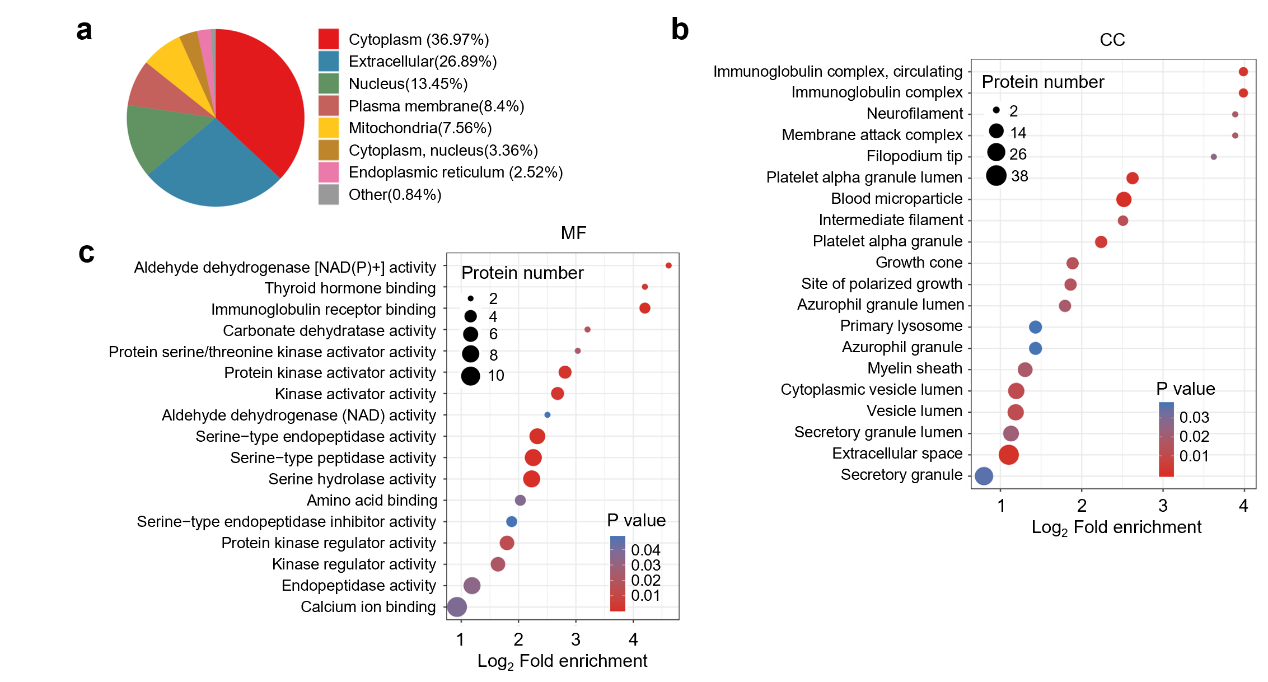


**Supplementary Fig. 2 Function enrichment analysis of DAPs in VSVO and MDVO**

**a** The subcellular location of DAPS among VSVO and MDVO specimens. Bubble diagram representing the DAPs of among VSVO and MDVO enriched in the (**b**) cellular components and (**c**) molecular functions, respectively.


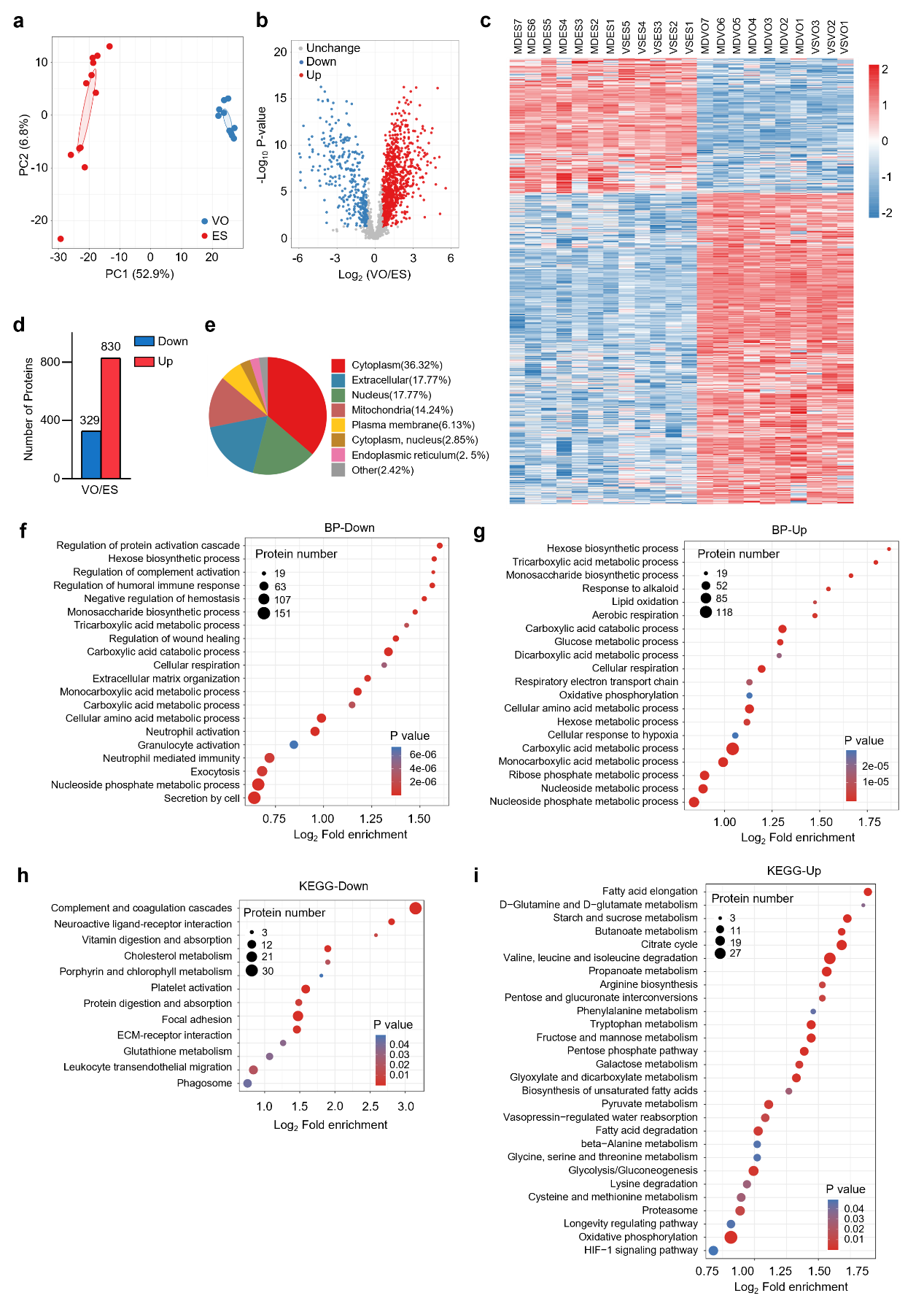


**Supplementary Fig. 3 Hierarchical clustering and function enrichment analysis of DAPs in the VO and ES**

**a** Protein quantitative principal component analysis (PCA) results for ES and VO in patients with VS and MD all samples. **b** Volcano plot showing differences in protein expression for all proteins identified in ES and VO samples (red, upregulated proteins; blue, downregulated proteins; grey, unchanged proteins). **c** Heatmap showing the DAPs in ES and VO samples. **d** Histogram representing the distribution of DAPs in ES and VO samples. **e** The subcellular location of DAPs in VO and ES samples. Bubble diagrams illustrating the enrichment analysis of (**f, h**) downregulated and (**g, i**) upregulated DAPs enriched in the biological process category and KEGG.

**
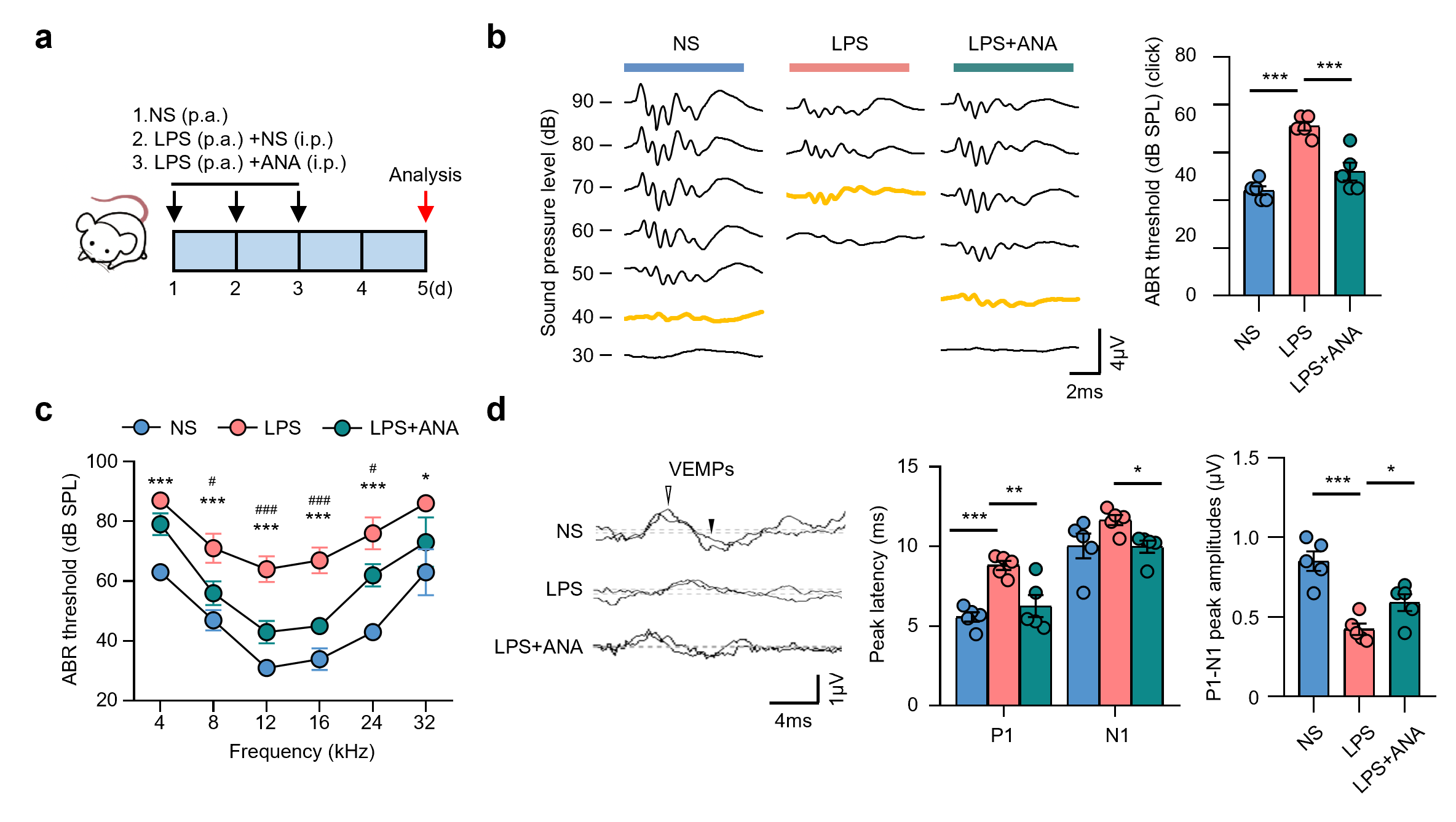
**

**Supplementary Fig. 4 Anakinra alleviates the phenotype of EH mice.**

**a** Mice were subjected to the IL1-receptor antagonist anakinra (10 mg/kg, i.p.) or an equivalent NS 30 minutes prior to LPS (10mg/kg, p.a.) challenge for 3 consecutive days, then analyzed at 5 days. **b** Representative serial ABR wave recordings and thresholds in response to click sounds (n=6). **c** ABR thresholds in response to tone pip across all frequencies tested (4, 8, 12, 16, 24, and 32 kHz) (n=5). * significant difference compared with NS and LPS; # significant difference compared with LPS and LPS+ANA. **d** Representative click-evoked VEMP waves, P1-N1 peak amplitudes, and the P1 (white triangle) and N1 (black triangle) peak latencies of VEMPs at 100 dB nHL (n=5). * or # p < 0.05, ** or ## p < 0.01, *** or ### p < 0.001.
