# Supplementary figures and images for: IL-1β promotes glutamate excitotoxicity: indications for the link between inflammatory and synaptic vesicle cycle in Ménière’s disease
Source: Cell Death Discov. 2024 Nov 20;10:476. doi: 10.1038/s41420-024-02246-2 (PMC11579495; doi:10.1038/s41420-024-02246-2)

Figure 5b


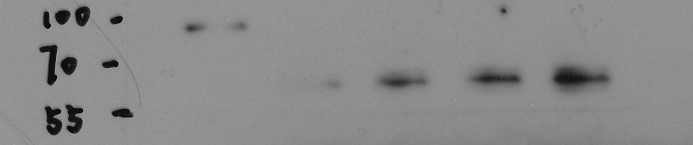

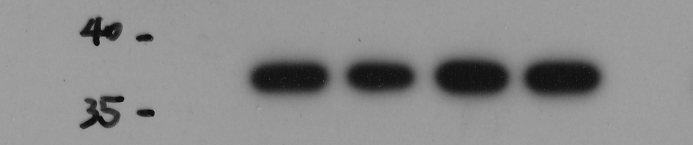

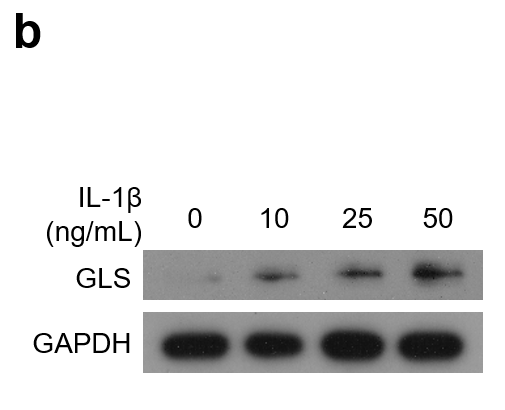


Figure 5e


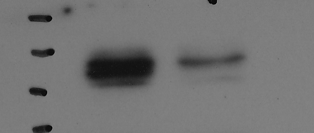


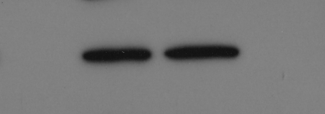


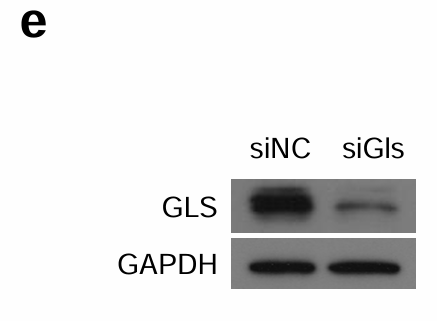

Supplement: Supplementary file 7 — original western blots [file 41420_2024_2246_MOESM7_ESM.docx]
